# Supplementary material for: Monitoring the Circulation of SARS-CoV-2 Variants by Genomic Analysis of Wastewater in Marseille, South-East France
Source: Pathogens. 2021 Aug 17;10(8):1042. doi: 10.3390/pathogens10081042 (PMC8401729; doi:10.3390/pathogens10081042)
Supplement: Supplementary file 1 [file pathogens-10-01042-s001.zip › Table S1.pdf]

Table S1: Evaluation of efficacity of Bio-T Kit® SARS-CoV-2 UK & N501Y variants (Biosellal, BIOTK125) for the detection of variants

**Anonymous sample name**	**Ct Gene E**	**Ct Gene S with N501Y mutation**	**Ct Gene S with Δ69-70 deletion**	**Conclusion Bio-T Kit**	IHU-MI	Classification according to NextClade	WHO label	Pango lineage
1	13,0	NI	NI	Other SARS-CoV-2 variants	3	20B		N.2
2	12,1	NI	NI	Other SARS-CoV-2 variants	2098	20A		B.1.160
3	13,9	NI	NI	Other SARS-CoV-2 variants	669	20A		B.1.36.10
4	11,9	14,2	NI	SA-BR-Marseille N501Y variants	3191	20J (Brazilian)	Gamma	P.1
5	12,7	14,1	NI	SA-BR-Marseille N501Y variants	3368	20J (Brazilian)	Gamma	P.1
6	11,4	13,2	NI	SA-BR-Marseille N501Y variants	3217	19B (Marseille 501)		A.27
7	14,1	NI	NI	Other SARS-CoV-2 variants	2128	20A.EU2		B.1.160
8	13,8	NI	NI	Other SARS-CoV-2 variants	3197	20A.EU2		B.1.160
9	13,3	NI	NI	Other SARS-CoV-2 variants	2123	20A		B.1.416
10	14,6	16,3	15,6	UK variants	3428	20I (UK)	Alpha	B.1.1.7
11	12,7	NI	NI	Other SARS-CoV-2 variants	2792	20A.EU2		B.1.160
12	14,9	NI	NI	Other SARS-CoV-2 variants	3203	20A.EU2		B.1.160
13	13,3	15,0	14,6	UK variants	3510	20I (UK)	Alpha	B.1.1.7
14	13,6	NI	NI	Other SARS-CoV-2 variants	2096	20A.EU2		B.1.160
15	14,2	NI	NI	Other SARS-CoV-2 variants	3563	21D (Marseille484K.V3-21D)	Eta	B.1.525
16	14,2	NI	NI	Other SARS-CoV-2 variants	3239	20B (Marseille484K.V1-20B)		R.1
17	14,5	16,1	NI	SA-BR-Marseille N501Y variants	3228	20H (South African)	Beta	B.1.351
18	11,7	NI	NI	Other SARS-CoV-2 variants	2129	20A.EU2		B.1.160
19	12,4	NI	NI	Other SARS-CoV-2 variants	2137	20A		B.1.160
20	12,2	NI	NI	Other SARS-CoV-2 variants	845	20C		B.1
21	10,8	12,7	NI	SA-BR-Marseille N501Y variants	3224	19B (Marseille 501)		A.27
22	12,5	NI	NI	Other SARS-CoV-2 variants	2178	20A		B.1.416
23	14,1	16,0	NI	SA-BR-Marseille N501Y variants	3227	20H (South African)	Beta	B.1.351

24	13,4	NI	NI	Other SARS-CoV-2 variants	2122	20A		B.1.416
25	14,2	15,1	15,1	UK variants	3505	20I (UK)	Alpha	B.1.1.7
26	12,8	14,7	NI	SA-BR-Marseille N501Y variants	3147	20H (South African)	Beta	B.1.351
27	13,7	15,6	15,1	UK variants	3076	20I (UK)	Alpha	B.1.1.7
28	13,3	15,3	NI	SA-BR-Marseille N501Y variants	3242	20H (South African)	Beta	B.1.351
29	11,3	NI	NI	Other SARS-CoV-2 variants	2514	20C		B.1.367
30	12,2	NI	NI	Other SARS-CoV-2 variants	3179	20A.EU2		B.1.160
31	14,4	NI	NI	Other SARS-CoV-2 variants	3564	21D (Marseille484K.V3-21D)	Eta	B.1.525
