# Supplementary figures and images for: Monitoring the Circulation of SARS-CoV-2 Variants by Genomic Analysis of Wastewater in Marseille, South-East France
Source: Pathogens. 2021 Aug 17;10(8):1042. doi: 10.3390/pathogens10081042 (PMC8401729; doi:10.3390/pathogens10081042)

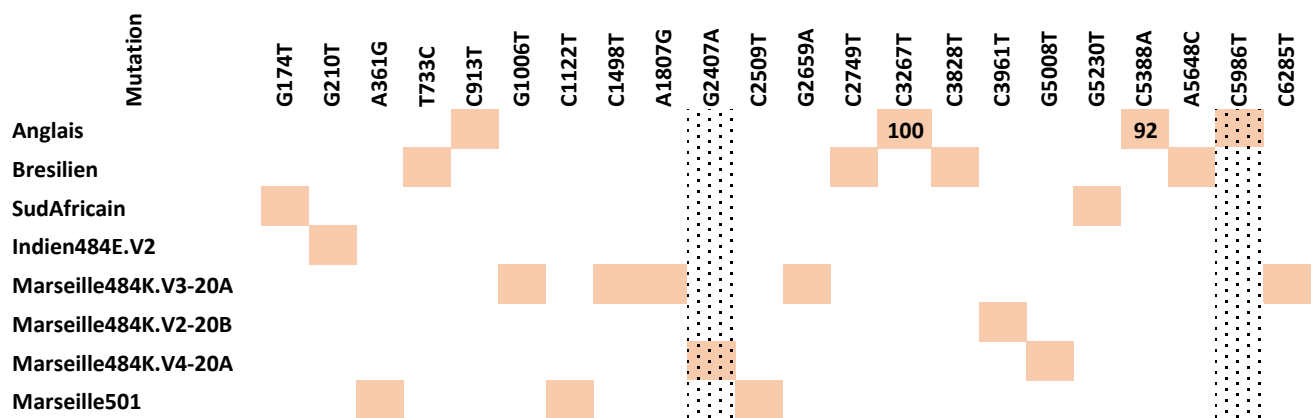

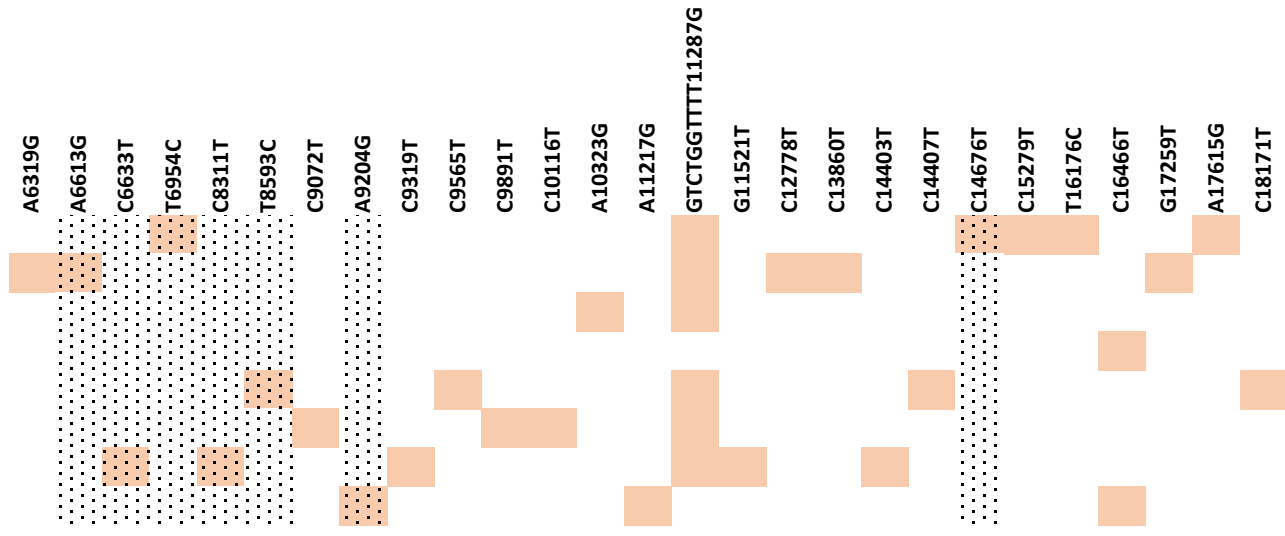

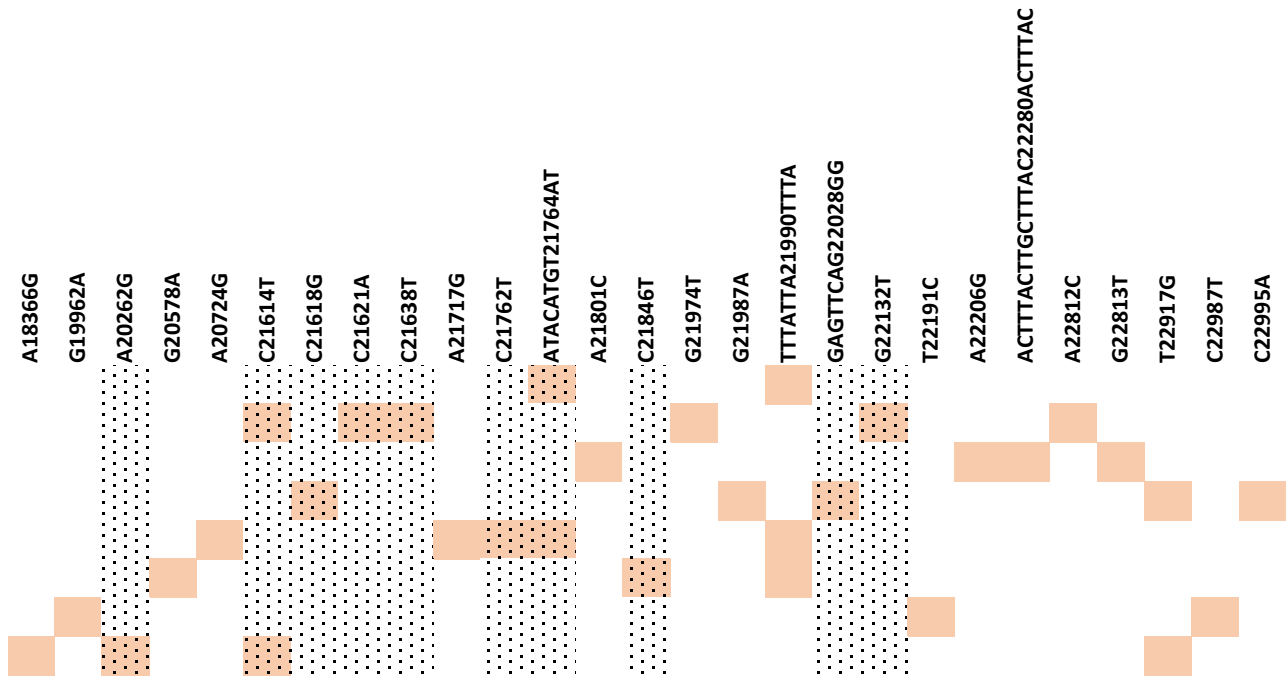

[illegible]

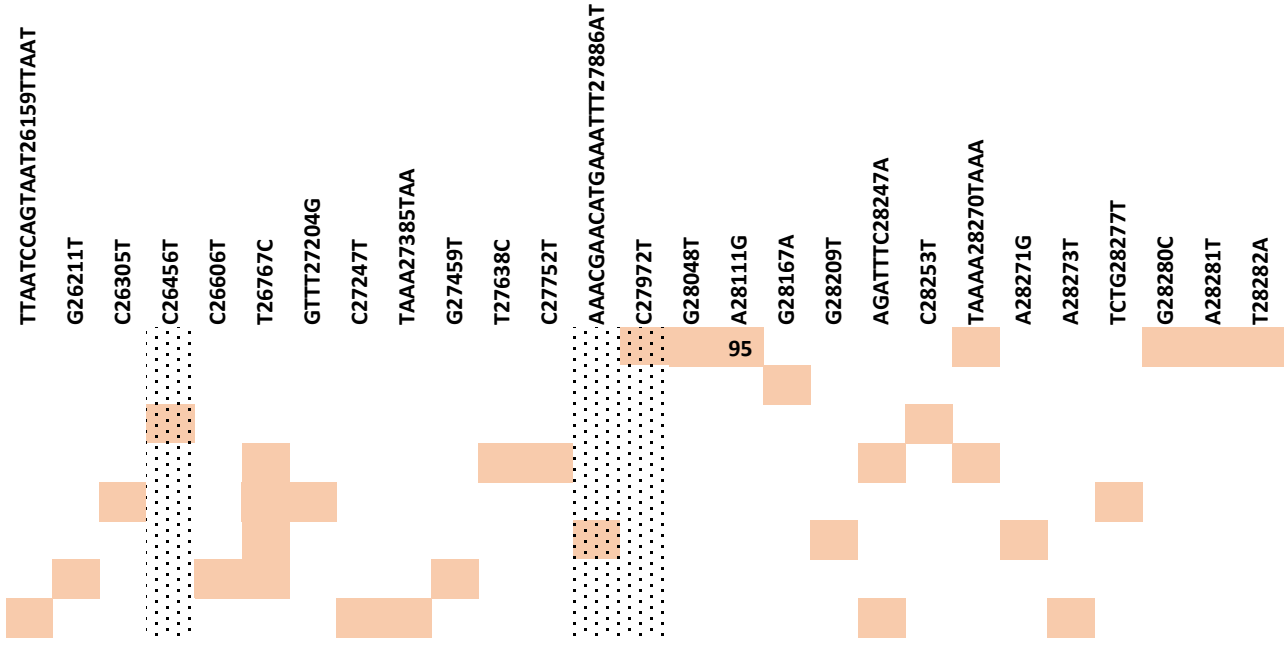

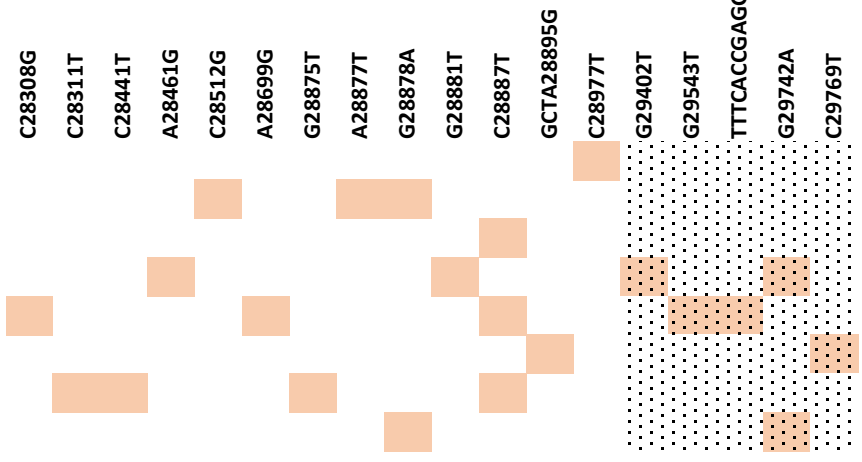

Supplement: Supplementary file 1 [file pathogens-10-01042-s001.zip › Table S2-1 Matrix showing the pattern mutations for RS sample.pdf]
